# Electrical and Optical Properties of a Transparent Conductive ITO/Ga_2_O_3_/Ag/Ga_2_O_3_ Multilayer for Ultraviolet Light-Emitting Diodes

**DOI:** 10.3390/nano9030403

**Published:** 2019-03-10

**Authors:** Siwei Liang, Quanbin Zhou, Xianhui Li, Ming Zhong, Hong Wang

**Affiliations:** 1 Engineering Research Center for Optoelectronics of Guangdong Province, School of Physics and Optoelectronics, South China University of Technology, Guangzhou 510640, China; phswliang@foxmail.com (S.L.); zhouquanbin86@163.com (Q.Z.); lxh0303@outlook.com (X.L.); jzryzhong@foxmail.com (M.Z.); 2 Engineering Laboratory for Wide Band gap Semiconductor Materials and Devices of Guangdong Province, School of Electronics and Information Engineering, South China University of Technology, Guangzhou 510640, China; 3 Zhongshan Institute of Modern Industrial Technology, South China University of Technology, Zhongshan 528437, China

**Keywords:** Ga_2_O_3_, transparent conductive electrode, UV LEDs, transmittance, sheet resistance

## Abstract

We fabricated an indium tin oxide (ITO)/Ga_2_O_3_/Ag/Ga_2_O_3_ multilayer as a transparent conductive electrode for ultraviolet light-emitting diodes (UV LEDs). The electrical and optical properties of the multilayer were improved by optimizing the annealing temperature of the ITO contact layer and the whole ITO/Ga_2_O_3_/Ag/Ga_2_O_3_ multilayer, and the thickness of the ITO contact layer and Ag metal layer. After optimization, the sheet resistance and transmittance of the ITO/Ga_2_O_3_/Ag/Ga_2_O_3_ multilayer was 3.43 Ω/sq and 86.4% at 335 nm, respectively. The ITO/Ga_2_O_3_/Ag/Ga_2_O_3_ multilayer also exhibited a good ohmic contact characteristic with a specific contact resistance of 1.45×10^−3^ Ω·cm^2^. These results show that the proposed ITO/Ga_2_O_3_/Ag/Ga_2_O_3_ multilayer is a promising alternative as a *p*-type electrode for UV LEDs.

## 1. Introduction

The III-nitride-based ultraviolet light-emitting diodes (UV LEDs) have broad application prospects in sterilization, UV curing, biochemical detection, phototherapy, and special lighting. Compared with traditional mercury lamps, UV LEDs have many advantages, such as being environmentally friendly, compact, having a long lifetime, and low voltage [1]. For blue and green LEDs, indium tin oxide (ITO) is the most widely used transparent conductive electrode (TCE) because of its good conductivity and high transmittance in visible range [2,3,4]. However, ITO is not the best choice for UV LEDs because the transmittance of ITO drops sharply in the ultraviolet region due to its narrow optical band gap [5]. Different methods were reported to solve this problem, such as metal-doped ITO [6,7,8], fluorine-doped ITO [5], and ITO/metal/ITO multilayers [9,10]. Although a transmittance of more than 90% has been achieved at wavelengths above 380 nm, the light absorption of ITO in the deep ultraviolet region is still severe.

In recent years, researchers have proposed to replace ITO with ZnO-based [11,12,13], Ga_2_O_3_-based [14,15,16], and graphene-based [17,18,19,20] TCEs in UV LEDs. Ga_2_O_3_, as an alternative to ITO, has a wider band gap (4.9 eV) and thus less absorption in ultraviolet light than ITO [21,22]. However, Ga_2_O_3_ exhibits insulating properties because its electrical conductivity is poor. Also, it is difficult to dope Ga_2_O_3_ with other metals. This mean the preparation of conductive β-Ga_2_O_3_ requires a complicated environment and a high temperature [23]. Therefore, to improve the performance of UV LEDs which use Ga_2_O_3_ as TCE, many methods have been reported to enhance the conductivity of Ga_2_O_3_ as well as the transmittance. It was reported that a Ga_2_O_3_ film with good conductivity (50 Ω^−1^·cm^−1^) was achieved by doping Si [24]. But the conductivity is still too low to be used as TCE in UV LEDs. Liu et al. first deposited Ga_2_O_3_/ITO films using magnetron sputtering and achieved a transmittance of 77.6% at 280 nm, but a large sheet resistance of 323 Ω/sq compared to ITO [25]. Li et al. concluded that different substrate temperatures while depositing ITO/Ga_2_O_3_ films can change the optical band gap and finally obtained the bi-layer films with a transmittance of 78.97% at 300 nm, but a sheet resistance of 373.3 Ω/sq [26]. Kim et al. also optimized the ITO/Ga_2_O_3_ multilayer layer by adjusting the growth temperature and the post-annealing temperature. The films with a sheet resistance of 49 Ω/sq and a transmittance of 93.8% at 405 nm were achieved after optimization [16]. Woo et al. fabricated an Ag-doped Ga_2_O_3_ layer with a sheet resistance of 42 Ω/sq after annealing in air at 550 °C and realized a transmittance of 83% at 385 nm [14]. In order to improve the ohmic contact with *p*-GaN, they deposited a 3-nm thick Ni before depositing the Ag-doped Ga_2_O_3_ layer [15].

In a previous study, we demonstrated that the ITO/Ga_2_O_3_/Ag/Ga_2_O_3_ multilayer has advantages in conductivity and transmittance for UV LEDs compared to ITO [27]. The ITO/Ga_2_O_3_/Ag/Ga_2_O_3_ multilayer consists of an ITO contact layer, a Ga_2_O_3_ layer, an Ag metal layer, and another Ga_2_O_3_ layer, which are deposited in sequence. The ITO contact layer is used to improve the ohmic contact between Ga_2_O_3_ and *p*-GaN in AlGaN-based UV LED epitaxial wafers. However, the process of ITO/Ga_2_O_3_/Ag/Ga_2_O_3_ multilayer still needs to be optimized in order to further enhance the performance of UV LEDs which use Ga_2_O_3_ as a TCE. In this paper, we systematically studied the effect of the thickness of the Ag metal layer and the ITO contact layer on the sheet resistance and transmittance. Different annealing temperatures of the ITO contact layer and the whole ITO/Ga_2_O_3_/Ag/Ga_2_O_3_ multilayer were also compared respectively. Furthermore, the ITO/Ga_2_O_3_/Ag/Ga_2_O_3_ multilayer was compared with the conventional ITO in the sheet resistance, transmittance and the specific contact resistance.

## 2. Materials and Methods

**Figure 1:**
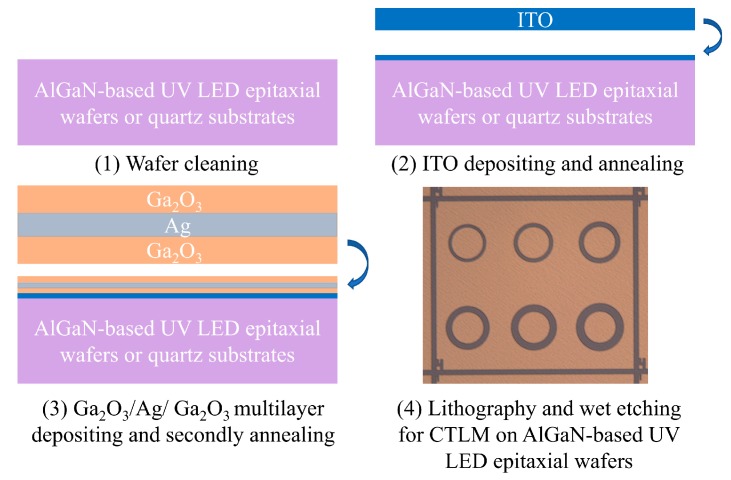
The procedures of making the indium tin oxide (ITO)/Ga_2_O_3_/Ag/Ga_2_O_3_ multilayer and optical micrograph of contact surface morphologies on circular transmission line model (CTLM) patterns. The UV LED is the abbreviation of ultraviolet light-emitting diode.

We prepared a series of ITO/Ga_2_O_3_/Ag/Ga_2_O_3_ multilayers with different thicknesses of ITO contact layer and Ag metal layer as well as different annealing temperatures on quartz substrates and AlGaN-based UV LED epitaxial wafers. The AlGaN-based UV LED structures used in this study were grown on a *c*-plane sapphire substrate by metalorganic chemical vapor deposition (MOCVD). The epitaxial structure consists of a 2.5-μm-thick undoped GaN layer, a 2-μm-thick *n*-GaN layer, a 150-nm-thick AlGaN-based multiple quantum wells (MQWs) active layer, a 20-nm-thick AlGaN electron blocking layer, and a 200-nm-thick *p*-GaN layer. The detailed processes of sample preparation are as below. As shown in step (1) of Figure 1, the quartz substrates and the AlGaN-based UV LED epitaxial wafers were first ultrasonically cleaned in acetone and isopropanol, then rinsed in deionized water and blown dry in nitrogen ambient. Next, as shown in step (2) of Figure 1, we deposited an ITO contact layer on quartz substrates or LED epitaxial wafers using electron-beam evaporation and pure ITO targets (In_2_O_3_:SnO_2_ = 90:10 wt.%). Then, the samples were annealed in the rapid thermal annealing (RTA) system at specific temperatures for 1 min in N_2_/O_2_ mixture ambient (N_2_:O_2_ = 200:35 sccm). After that, as shown in step (3) of Figure 1, we deposited a Ga_2_O_3_ layer, an Ag metal layer, and another Ga_2_O_3_ layer in sequence via magnetron sputtering systems. The Ga_2_O_3_ layers were deposited by radio frequency (RF) magnetron sputtering of Ga_2_O_3_ (purity 99.99%) ceramic targets and the Ag metal layer was deposited by direct current (DC) magnetron sputtering of Ag targets. The thickness of Ga_2_O_3_ layers was 15 nm for all samples in this study. Finally, the whole ITO/Ga_2_O_3_/Ag/Ga_2_O_3_ multilayer was annealed again in the RTA system at specific temperatures for 1 min in N_2_/O_2_ mixture ambient (N_2_:O_2_ = 200:35 sccm).

We measured the sheet resistance and the transmittance of the multilayers on the quartz substrates. The sheet resistance was measured by four-point probe methods. The transmittance was measured by the UV/visible spectrophotometer after using the blank quartz substrate to calibrate the baseline, which meant the transmittance of blank quartz substrates was 100%. The specific contact resistance was measured through the circular transmission line model (CTLM) patterns processed by the standard photolithographic technique on LED epitaxial wafers. The optical micrograph of contact surface morphologies on CTLM patterns are shown in step (4) of Figure 1. The CTLM patterns consist of six circles which have the same inner circle radius of 75 μm. The spacings between the inner circle and the outer circle are 15, 20, 25, 30, 35, and 40 μm. The current–voltage (*I*-*V*) characteristic curves shown below are measured between the inner circle and the outer region separated by 15 μm using the *keysight B1505A* measurement system.

A traditional 60-nm ITO thin film on the quartz substrate and the AlGaN-based UV LED epitaxial wafer were prepared as reference and annealed using our optimized conditions at 550 °C for 2 min in N_2_/O_2_ mixture ambient (N_2_:O_2_ = 200:35 sccm). The procedures were similar to ITO/Ga_2_O_3_/Ag/Ga_2_O_3_ multilayers as shown in step (1), (2), and (4) of Figure 1.

## 3. Results and Discussion

**Figure 2:**
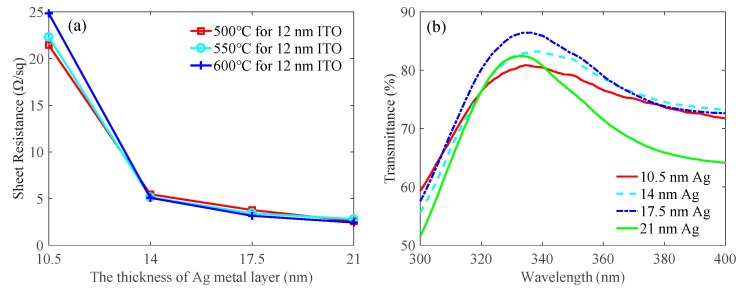
(**a**) Effects of the thickness of the Ag metal layer and annealing temperature of ITO contact layer on the sheet resistance of the ITO/Ga_2_O_3_/Ag/Ga_2_O_3_ multilayer when the annealing temperature of the whole ITO/Ga_2_O_3_/Ag/Ga_2_O_3_ multilayer was 600 °C. (**b**) Effects of the thickness of the Ag metal layer on transmittance of the ITO/Ga_2_O_3_/Ag/Ga_2_O_3_ multilayer when the annealing temperatures of the ITO contact layer and the whole ITO/Ga_2_O_3_/Ag/Ga_2_O_3_ multilayer were 550 °C and 600 °C, respectively.

Firstly, we studied how the thickness of Ag metal layer affected the sheet resistance and transmittance of the ITO/Ga_2_O_3_/Ag/Ga_2_O_3_ multilayer and determined the optimal thickness. As shown in Table 1, the samples were divided into four groups. In each group, we first deposited a 12-nm-thick ITO contact layer on quartz substrates and annealed at 500 °C to 600 °C for 1 min in N_2_/O_2_ mixture ambient (N_2_:O_2_ = 200:35 sccm). Then, the Ga_2_O_3_/Ag/Ga_2_O_3_ multilayers were deposited on the ITO contact layer. The thickness of each Ga_2_O_3_ layer was 15 nm and the thickness of the Ag metal layer in each group was designed as 10.5 nm, 14 nm, 17.5 nm, and 21 nm, respectively. Next, the whole ITO/Ga_2_O_3_/Ag/Ga_2_O_3_ multilayer was annealed at 600 °C for 1min in N_2_/O_2_ mixture ambient (N_2_:O_2_ = 200:35 sccm). Figure 2a shows that the sheet resistance of the ITO/Ga_2_O_3_/Ag/Ga_2_O_3_ multilayer decreased gradually as the thickness of the Ag metal layer increased. The sheet resistance of the multilayer decreased abruptly when the thickness of Ag metal layer increased from 10.5 nm to 14 nm and then decreased slowly when the thickness of Ag metal layer continued to increase. Similar results were seen in the previous research using the Oxide/Ag/Oxide concept, such as ITO/Ag/ITO multilayer [28] and ZnSnO_3_/Ag/ZnSnO_3_ multilayer [29]. The abrupt decrease in sheet resistance is attributed to the connections between Ag islands which develop a conductive path. Further decrease in sheet resistance is attributed to the conductive path through the thicker Ag metal layer between Ga_2_O_3_ layers. This shows that the thickness of the Ag metal layer embedded within Ga_2_O_3_ layers is important for spreading current. However, the transmittance increased first and then decreased when the thickness of Ag metal layer increased from 10.5 nm to 21 nm, as shown in Figure 2b. The transmittances were 80.8%, 83.0%, 86.4%, and 82.3% at 335 nm for the 10.5-nm-thick Ag, 14-nm-thick Ag, 17.5-nm-thick Ag, and 21-nm-thick Ag, respectively. We have only showed the results of those samples whose ITO are annealed at 550 °C. Other samples with ITO annealed at 500 °C and 600 °C also showed the same trend. When the thickness of the Ag metal layer was 10.5 nm, the lowest transmittance was observed, which may be due to the strong scattered light on the Ag islands. With increase of the thickness of the Ag metal layer, the Ag islands are connected, which weakens the scattered light and therefore increases the transmittance. However, as the thickness of the Ag metal layer continued to increase, the transmittance decreased, caused by the light reflection on the Ag metal layer. The highest transmittance at a particular Ag thickness may be also due to the antireflection effects, as suggested by Fan el al. [30]. Therefore, we chose the optimal thickness of the Ag metal layer as 17.5 nm for the higher transmittance and smaller sheet resistance.

**Figure 3:**
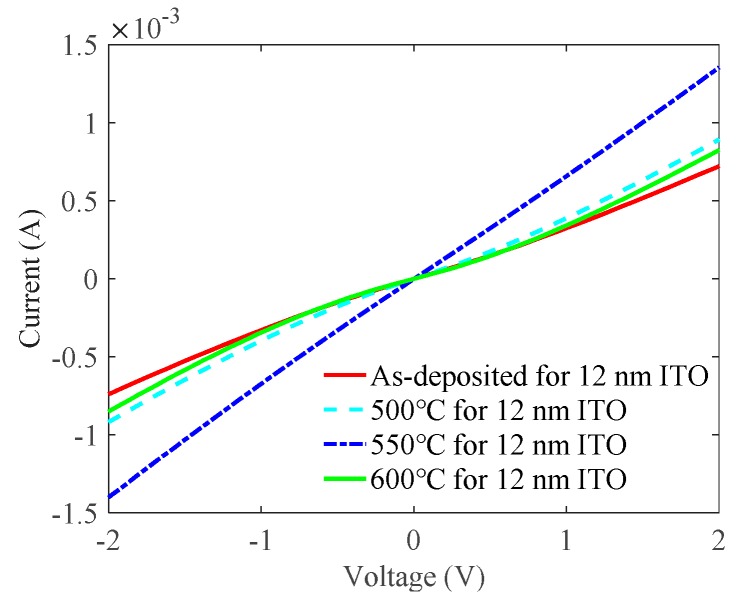
Current-voltage (*I*-*V*) characteristic curves of the multilayer with different annealing temperatures of the ITO contact layer when the thickness of the Ag metal layer was 17.5 nm and the annealing temperature of the whole ITO/Ga_2_O_3_/Ag/Ga_2_O_3_ multilayer was 600 °C.

Figure 2a demonstrates the relationship between the annealing temperature of the ITO contact layer and the sheet resistance of the multilayer. As shown in Figure 2a, the annealing temperature of the ITO contact layer had little effect on the sheet resistance, especially when the thickness of the Ag metal layer increased. Not shown here, the annealing temperature of the ITO contact layer also had an insignificant effect on the transmittance. To demonstrate how the annealing temperature of the ITO contact layer affected the ohmic contact between ITO and *p*-GaN, we prepared the ITO/Ga_2_O_3_/Ag/Ga_2_O_3_ multilayer on UV LED epitaxial wafers according to the experiment parameters of group 3 in Table 1. Figure 3 plots the *I*-*V* characteristic curves of the ITO/Ga_2_O_3_/Ag/Ga_2_O_3_ multilayer on LED epitaxial wafers with different annealing temperatures of the ITO contact layer when the thickness of the Ag metal layer was 17.5 nm and the annealing temperature of the whole ITO/Ga_2_O_3_/Ag/Ga_2_O_3_ multilayer was 600 °C. The *I*-*V* characteristic of the as-deposited sample with a second annealing process is also shown for comparison. The slope of the *I*-*V* curve increases first and then decreases as the annealing temperature of the ITO contact layer increases. The specific contact resistances calculated through CLTM are 2.17 × 10^−2^, 7.52 × 10^−3^, 1.45 × 10^−3^, and 9.01 × 10^−3^ Ω·cm^2^ for the as-deposited, 500 °C, 550 °C, and 600 °C samples, respectively. The multilayer with the ITO annealed at 550 °C exhibited the best ohmic contact with *p*-GaN among these samples. Therefore, we chose the optimal annealing temperature of the ITO contact layer as 550 °C for better ohmic contact.

**Figure 4:**
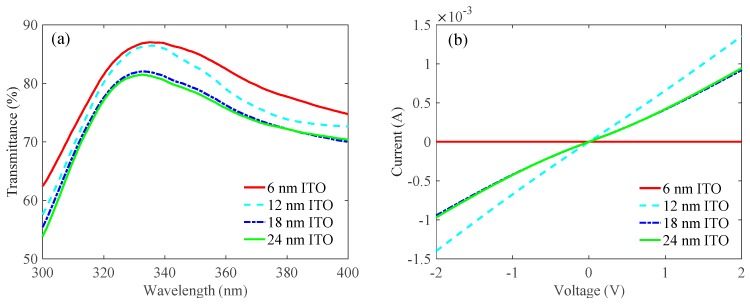
Effects of the thickness of the ITO contact layer on (**a**) transmittances and (**b**) *I*-*V* characteristic curves of the ITO/Ga_2_O_3_/Ag/Ga_2_O_3_ multilayer when the thickness of the Ag metal layer was 17.5 nm and the annealing temperature of the ITO contact layer and the whole ITO/Ga_2_O_3_/Ag/Ga_2_O_3_ multilayer were 550 °C and 600 °C, respectively.

After determining the optimal thickness of the Ag metal layer and the annealing temperature of the ITO contact layer, we investigated the optimal thickness of the ITO contact layer. As shown in Table 2, a 6-nm-thick ITO, 12-nm-thick ITO, 18-nm-thick ITO, and 24-nm-thick ITO were first deposited on the quartz substrates and the LED epitaxial wafers, respectively. Then, all samples were annealed at 550 °C for 1 min in N_2_/O_2_ mixture ambient (N_2_:O_2_ = 200:35 sccm). After that, we deposited a 15-nm-thick Ga_2_O_3_ layer, a 17.5-nm-thick Ag metal layer, and another 15-nm-thick Ga_2_O_3_ layer in sequence and annealed at 600 °C for 1 min in N_2_/O_2_ mixture ambient (N_2_:O_2_ = 200:35 sccm). Figure 4a shows the transmittance spectra measured for the ITO/Ga_2_O_3_/Ag/Ga_2_O_3_ multilayer on quartz substrates as a function of different thicknesses of the ITO contact layer. The decrease in transmittance with increasing ITO thickness is attributed to the absorption of light by ITO. The multilayers exhibited transmittances of 87.0%, 86.4%, 81.9%, and 81.3% at 335 nm for ITO thicknesses of 6 nm, 12 nm, 18 nm, and 24 nm, respectively. Figure 4b shows the *I*-*V* characteristic curves of ITO/Ga_2_O_3_/Ag/Ga_2_O_3_ multilayer on LED epitaxial wafers as a function of different thicknesses of ITO contact layers. However, the multilayer with the ITO thickness of 6 nm exhibited insulating *I*-*V* characteristic. This is because the ITO was too thin to develop a uniform film and was deposited as discrete nanoparticles. The specific contact resistances calculated from the *I*-*V* characteristics were 1.45 × 10^−3^, 7.12 × 10^−3^, and 6.84 × 10^−3^ Ω·cm^2^ for the 12-nm-thick, 18-nm-thick, and 24-nm-thick samples, respectively. Therefore, we chose the optimal thickness of the ITO contact layer as 12 nm for the higher transmittance and better ohmic contact.

**Figure 5:**
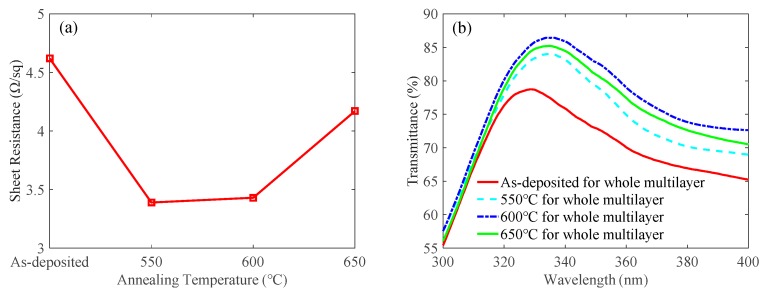
Effects of the annealing temperature of the whole ITO/Ga_2_O_3_/Ag/Ga_2_O_3_ multilayer on (**a**) sheet resistance and (**b**) transmittance when the thickness of the ITO contact layer was 12 nm, the annealing temperature of the ITO contact layer was 550 °C, and the thickness of the Ag metal layer was 17.5 nm.

In addition, we investigated the optimal annealing temperature of the whole ITO/Ga_2_O_3_/Ag/Ga_2_O_3_ multilayer. As shown in Table 3, we first deposited a 12-nm-thick ITO contact layer and annealed at 550 °C for 1 min in N_2_/O_2_ mixture ambient (N_2_:O_2_ = 200:35 sccm) on quartz substrates and UV LED epitaxial wafers. After that, a 15-nm-thick Ga_2_O_3_ film, a 17.5-nm-thick Ag metal layer and another 15-nm-thick Ga_2_O_3_ film were deposited on the ITO contact layer. Finally, the whole multilayers were annealed at different temperatures from 550 °C to 650 °C. The as-deposited sample without a second annealing process was also prepared for comparison. As shown in Figure 5a, the sheet resistances were 4.62 Ω/sq, 3.39 Ω/sq, 3.43 Ω/sq, and 4.17 Ω/sq for the as-deposited, 550 °C, 600 °C, and 650 °C samples, respectively. The secondly annealing process decreased the sheet resistance, which was attributed to the improved crystal quality. However, the sheet resistance increased when the annealing temperature was 650 °C. This may be attributed to the Ag agglomeration in the high annealing temperature. The multilayers with an Ag thickness of 10.5 nm, 14 nm, and 21 nm had the same trend, which are not shown here. Figure 5b shows the optical transmittance spectra measured on quartz substrates as a function of different annealing temperatures of the whole multilayer. The transmittance of the as-deposited multilayer was 77.4% at 335 nm. With the annealing temperature increasing, the transmittance increased first and reached 86.4% at 335 nm when the annealing temperature was 600 °C. This phenomenon is caused by the diffusion of Ag atoms into Ga_2_O_3_ layers, which leads to weaker light reflection by the Ag layer. After that, when the annealing temperature further rose to 650 °C, the transmittance decreased to 85.2%, probably due to the Ag agglomeration in the high annealing temperature [10].

**Figure 6:**
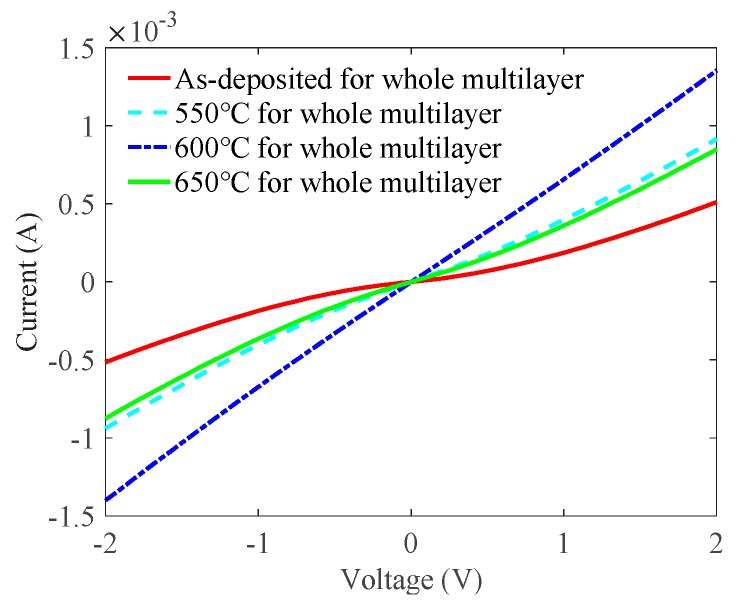
Effects of the annealing temperature of the whole ITO/Ga_2_O_3_/Ag/Ga_2_O_3_ multilayer on *I*-*V* characteristic curves of the ITO/Ga_2_O_3_/Ag/Ga_2_O_3_ multilayer when the thickness of the ITO contact layer was 12 nm, the annealing temperature of the ITO contact layer was 550 °C, and the thickness of the Ag metal layer was 17.5 nm.

Figure 6 shows the *I*-*V* characteristic curves on LED epitaxial wafers as a function of different annealing temperatures of the whole ITO/Ga_2_O_3_/Ag/Ga_2_O_3_ multilayer. The as-deposited multilayer showed nonlinear *I*-*V* characteristics with a low current of 0.52 mA at 2 V. On the other hand, thermally annealing the multilayer significantly improved its *I*-*V* characteristic. The samples annealed at 550 °C and 600 °C exhibited current values of 0.904 and 1.38 mA at 2 V, respectively. This indicates that the second annealing process influenced the ohmic contact between ITO and *p*-GaN. When the annealing temperature reached 650 °C, the current values decreased to 0.87 mA at 2 V. This was because the high temperature worsened the ohmic contact between ITO and *p*-GaN. The specific contact resistances calculated from the *I*-*V* characteristic were 5.19 × 10^−2^, 6.10 × 10^−3^, 1.45 × 10^−3^, and 1.02 × 10^−2^ Ω·cm^2^ for the as-deposited 550 °C, 600 °C, and 650 °C samples, respectively. Therefore, we chose the optimal annealing temperature of the whole ITO/Ga_2_O_3_/Ag/Ga_2_O_3_ multilayer as 600 °C for higher transmittance and better ohmic contact.

**Figure 7:**
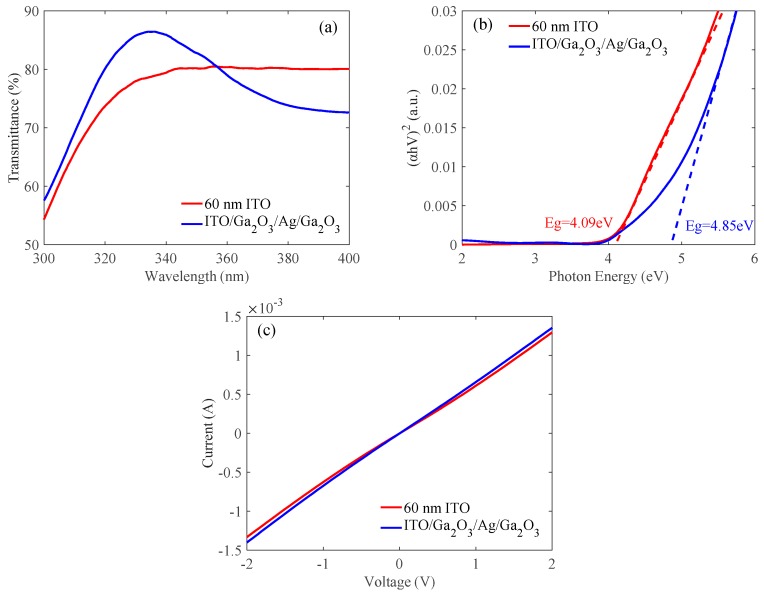
(**a**) Transmittances, (**b**) Energy bandgaps, and (**c**) *I*-*V* characteristic curves of the 60-nm ITO and the ITO/Ga_2_O_3_/Ag/Ga_2_O_3_ multilayer.

As reference, a traditional 60-nm-thick ITO was deposited on the quartz substrate and the UV LED epitaxial wafer to compare with the optimized ITO (12 nm)/Ga_2_O_3_ (15 nm)/Ag (17.5 nm)/Ga_2_O_3_ (15 nm) multilayer. The 60-nm-thick ITO was annealed at our optimized condition of 550 °C for 2 min in N_2_/O_2_ mixture ambient (N_2_:O_2_ = 200:35 sccm). Figure 7a shows the transmittances of the 60-nm ITO and the ITO/Ga_2_O_3_/Ag/Ga_2_O_3_ multilayer. As shown in Table 4, the transmittances were 78.7% and 86.4% at 335 nm for the 60-nm ITO and the ITO/Ga_2_O_3_/Ag/Ga_2_O_3_ multilayer, respectively. To further understand the origin of this result, the optical bandgap energy, *E_g_*, of the 60-nm ITO and the ITO/Ga_2_O_3_/Ag/Ga_2_O_3_ multilayer was calculated, respectively. The *E_g_* was estimated from the relation between (*αhv*)^2^ and *hv* according to Equations (1–3) as follows:

(1) $$\alpha hv=B{\left(hv-E_{g}\right)}^{1/2},$$

(2) $$hv=\frac{hc}{\lambda_{i}},$$

(3) $$\alpha =\frac{1}{d}ln\left(\frac{1}{T}\right),$$

where *α* is the light absorption coefficient, *hv* is the photon energy, *h* is planck constant bright, *c* is the light speed, *B* is a constant of direct transition, *λ_i_* is the wavelength, *d* is the thickness of films, and *T* is the transmittance [31,32,33]. The curves of (*αhv*)^2^ as a function of *hv* can be obtained as shown in Figure 7b and the *E_g_* can be estimated by extrapolating the linear section of (*αhv*)^2^ to the photon energy axis. The energy band gaps of the 60-nm ITO and the ITO/Ga_2_O_3_/Ag/Ga_2_O_3_ multilayer are estimated to be 4.09 and 4.85 eV, respectively. The wider band gap means less absorption of light in UV range. Therefore, the ITO/Ga_2_O_3_/Ag/Ga_2_O_3_ multilayer had larger transmittance than that of the 60-nm ITO. The ITO/Ga_2_O_3_/Ag/Ga_2_O_3_ multilayer also had a reduction in sheet resistance compared to the 60-nm ITO. As shown in Table 4, the sheet resistances were 51.55 Ω/sq and 3.43 Ω/sq for the 60-nm ITO and the ITO/Ga_2_O_3_/Ag/Ga_2_O_3_ multilayer, respectively. Figure 7c shows the *I*-*V* characteristic curves on LED epitaxial wafers of the 60-nm ITO and the ITO/Ga_2_O_3_/Ag/Ga_2_O_3_ multilayer. The specific contact resistances of the 60-nm ITO and the ITO/Ga_2_O_3_/Ag/Ga_2_O_3_ multilayer were 2.96 × 10^−3^ and 1.45 × 10^−3^ Ω·cm^2^, respectively. There was hardly any difference in specific contact resistance because the ohmic contact characteristic was mostly affected by the interface between ITO and *p*-GaN.

## 4. Conclusions and Further Work

In this paper, an ITO/Ga_2_O_3_/Ag/Ga_2_O_3_ multilayer with high conductivity and good transmittance was fabricated to be used as a transparent conductive electrode for UV LEDs. The influence of different Ag thicknesses, ITO thicknesses, and annealing temperatures of ITO and whole ITO/Ga_2_O_3_/Ag/Ga_2_O_3_ multilayers on electrical and optical properties of the ITO/Ga_2_O_3_/Ag/Ga_2_O_3_ multilayer were studied and analyzed systematically. Finally, an optimal structure of ITO (12 nm)/Ga_2_O_3_ (15 nm)/Ag (17.5 nm)/Ga_2_O_3_ (15 nm) with annealing temperatures of 550 °C for the ITO contact layer and 600 °C for the whole multilayer were obtained. The ITO/Ga_2_O_3_/Ag/Ga_2_O_3_ multilayer had a specific contact resistance of 1.45 × 10^−3^ Ω·cm^2^ and a highest transmittance of 86.4% at 335 nm. Compared with the traditional 60-nm ITO, the transmittance of the ITO/Ga_2_O_3_/Ag/Ga_2_O_3_ multilayer at 335 nm was higher due to the wider band gap of 4.85 eV. The multilayer also exhibited a good conductivity property with a sheet resistance of 3.43 Ω/sq, whereas the traditional 60-nm ITO was 51.55 Ω/sq. These results indicate that the ITO/Ga_2_O_3_/Ag/Ga_2_O_3_ multilayer shows great potential for application as a transparent conductive electrode for UV LEDs. In further work, we will fabricate UV LEDs using the ITO/Ga_2_O_3_/Ag/Ga_2_O_3_ multilayer as a *p*-type electrode.

## Figures and Tables

**Table 1:** The experimental design matrix for optimal thickness of the Ag metal layer and optimal annealing temperature of the ITO contact layer.

Group	The Thickness of the ITO Contact Layer	Annealing Temperature of the ITO Contact Layer	The Thickness of the Ag Metal Layer	Annealing Temperature of the Whole ITO/Ga_2_O_3_ (15 nm)/Ag/Ga_2_O_3_ (15 nm) Multilayer
1	12 nm	500 °C	10.5 nm	600 °C
		550 °C		
		600 °C		
2		500 °C	14 nm	
		550 °C		
		600 °C		
3		500 °C	17.5 nm	
		550 °C		
		600 °C		
4		500 °C	21 nm	
		550 °C		
		600 °C		

**Table 2:** The experimental design matrix for optimal thickness of the ITO contact layer.

Group	The Thickness of the ITO Contact Layer	Annealing Temperature of the ITO Contact Layer	The Thickness of the Ag Metal Layer	Annealing Temperature of the Whole ITO/Ga_2_O_3_ (15 nm)/Ag/Ga_2_O_3_ (15 nm) Multilayer
1	6 nm	550 °C	17.5 nm	600 °C
2	12 nm			
3	18 nm			
4	24 nm			

**Table 3:** The experimental design matrix for the optimal annealing temperature of the whole ITO/Ga_2_O_3_/Ag/Ga_2_O_3_ multilayer.

Group	The Thickness Of the ITO Contact Layer	Annealing Temperature of the ITO Contact Layer	The Thickness of the Ag Metal Layer	Annealing Temperature of the Whole ITO/Ga_2_O_3_ (15 nm)/Ag/Ga_2_O_3_ (15 nm) Multilayer
1	12 nm	550 °C	17.5 nm	As-deposited
2				550 °C
3				600 °C
4				650 °C

**Table 4:** Transmittance at 335 nm and sheet resistance of the 60-nm ITO and ITO/Ga_2_O_3_/Ag/Ga_2_O_3_ on quartz substrates.

Sample	Transmittance at 335 nm	Sheet Resistance	Specific Contact Resistances
**60-nm ITO**	78.7%	51.55 Ω/sq	2.96×10^−3^ Ω·cm^2^
**ITO/Ga_2_O_3_/Ag/Ga_2_O_3_**	86.4%	3.43 Ω/sq	1.45×10^−3^ Ω·cm^2^
